# Nickel bis­muth boride, Ni_23-*x*_Bi_*x*_B_6_ [*x* = 2.44 (1)]

**DOI:** 10.1107/S1600536811000894

**Published:** 2011-01-15

**Authors:** David Berthebaud, Akira Sato, Takao Mori

**Affiliations:** a National Institute for Materials Science, Namiki 1-1, Tsukuba, 305-0044 Japan

## Abstract

The τ-boride Ni_23-*x*_Bi_*x*_B_6_ [*x* = 2.44 (1)] adopts a ternary variant of the cubic Cr_23_C_6_-type structure, with Ni_8_ cubes and Ni_12_ cubocta­hedra arranged in a NaCl-type pattern. Two of the four independent metal sites (8*c*, 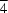
3*m* symmetry; 4*a*, *m* 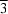 *m* symmetry) are occupied by a mixture of Ni and Bi atoms in a 0.106 (6):0.894 (6) and a 0.350 (7):0.650 (7) ratio, respectively.

## Related literature

For the structure of Cr_23_C_6_, see: Westgren (1933 ▶). For other examples of τ-borides, which have more than 80 representatives, see: Villars & Calvert (1985 ▶). For ternary ordered variants, see: Hillebrecht & Ade (1998 ▶) for *M*_20_ *M*′_3_B_6_; Veremchuk *et al.* (2009 ▶) for *M*_21_ *M*′_2_B_6_. For isotypic cobalt-containing solid solutions Co_23-_ *_x_M*’_*x*_B_6_, see: Kotzott *et al.* (2009 ▶).

## Experimental

### 

#### Crystal data

Ni_20.56_Bi_2.44_B_6_
*M* *_r_* = 1780.35
Cubic, 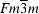

*a* = 10.575 (5) Å
*V* = 1182.6 (10) Å^3^
*Z* = 4
Mo *K*α radiation
μ = 67.81 mm^−1^
*T* = 293 K
0.10 × 0.08 × 0.06 mm

#### Data collection

Bruker SMART APEX CCD diffractometer
Absorption correction: multi-scan (*SADABS*; Bruker, 1999 ▶) *T*_min_ = 0.010, *T*_max_ = 0.067
6672 measured reflections
236 independent reflections
235 reflections with *I* > 2σ(*I*)
*R*_int_ = 0.044

#### Refinement

*R*[*F* ^2^ > 2σ(*F* ^2^)] = 0.020
*wR*(*F* ^2^) = 0.045
*S* = 1.16
236 reflections
15 parameters
Δρ_max_ = 2.18 e Å^−3^
Δρ_min_ = −2.22 e Å^−3^

### 

Data collection: *SMART* (Bruker, 1999 ▶); cell refinement: *SAINT-Plus* (Bruker, 1999 ▶); data reduction: *SAINT-Plus*; program(s) used to solve structure: *SIR2002* (Burla *et al.*, 2003 ▶); program(s) used to refine structure: *SHELXL97* (Sheldrick, 2008 ▶); molecular graphics: *DIAMOND* (Brandenburg, 2005 ▶); software used to prepare material for publication: *WinGX* (Farrugia, 1999 ▶).

## Supplementary Material

Crystal structure: contains datablocks global, I. DOI: 10.1107/S1600536811000894/mg2107sup1.cif

Structure factors: contains datablocks I. DOI: 10.1107/S1600536811000894/mg2107Isup2.hkl

Additional supplementary materials:  crystallographic information; 3D view; checkCIF report

## Figures and Tables

**Fig. 1.**
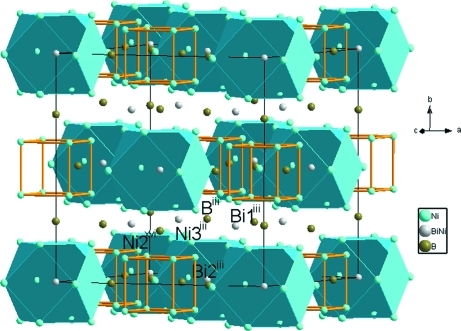
Structure of Ni23-xBixB6 highlighting the arrangement of Ni12 cuboctahedra (Ni3, 48 h) and Ni8 cubes (Ni2, 32f). Displacement ellipsoids are drawn at the 60% probability level. Symmetry codes are defined in the footnote of the table of geometric parameters.

## supplementary crystallographic information

### Comment

Ni_23-_*_x_*Bi*_x_*B_6_ belongs to a class of metal-rich compounds known as τ-borides, which are interesting ceramic materials. It is isostructural to numerous related *M*_20_*M*'_3_B_6_ and *M*_21_*M*'_2_B_6_ (*M* = 3*d* metal; *M*' = rare-earth, 4*d*, 5*d*, or main-group metal) phases, which adopt a ternary variant of the cubic Cr_23_C_6_-type structure (Westgren, 1933; Villars & Calvert, 1985; Hillebrecht & Ade, 1998; Veremchuk *et al.*, 2009). Of the four metal sites, two (32f and 48h) are occupied exclusively by Ni atoms giving a NaCl-type arrangement of Ni_8_ cubes and Ni_12_ cuboctahedra, and two (4a and 8c) are occupied by a mixture of Ni and Bi atoms, resulting in the composition Ni_20.56_Bi_2.44_B_6_ (Fig. 1). Mixed occupation of the 4a and 8c sites has also been previously reported in the Co-containing τ-borides Co_23-_*_x_M*'*_x_*B_6_ (Kotzott *et al.*, 2009).

### Experimental

A mixture of Ni, Bi, and B powders with nominal composition Ni_20_Bi_3_B_12_ was pressed into a pellet and placed in an alumina crucible. It was melted under Ar gas at 1473 K for 6 h, cooled at 20 K h^-1^ to 1273 K, and further cooled at 300 K h^-1^ to room temperature. The sample contained crystals of the title compound, in the presence of binary nickel borides, as revealed by powder X-ray diffraction analysis.

### Refinement

Several models involving mixed occupancy of Ni and Bi atoms, or vacancies (or both) within the metal sites were considered. We concluded that all sites are fully occupied, but two of them (4a and 8c) were disordered with a mixture of Ni and Bi atoms. Only an isotropic displacement parameter was refined for the B atom. The highest peak and the deepest hole in the final difference map are located at 2.12 and 0.57 Å from Ni3 and Bi1, respectively.

### Crystal data

**Table:**

B_6_Bi_2.44_Ni_20.56_	*D*_x_ = 9.999 Mg m^−^^3^
*M**_r_* = 1780.35	Mo *K*α radiation, λ = 0.71073 Å
Cubic, *F**m*3*m*	Cell parameters from 834 reflections
Hall symbol: -F 4 2 3	θ = 3.3–40.4°
*a* = 10.575 (5) Å	µ = 67.81 mm^−^^1^
*V* = 1182.6 (10) Å^3^	*T* = 293 K
*Z* = 4	Prism, grey
*F*(000) = 3231	0.10 × 0.08 × 0.06 mm

### Data collection

**Table:**

Bruker SMART APEX CCD diffractometer	235 reflections with *I* > 2σ(*I*)
graphite	*R*_int_ = 0.044
ω scans	θ_max_ = 40.4°, θ_min_ = 3.3°
Absorption correction: multi-scan (*SADABS*; Bruker, 1999)	*h* = −19→19
*T*_min_ = 0.010, *T*_max_ = 0.067	*k* = −19→19
6672 measured reflections	*l* = −17→19
236 independent reflections	

### Refinement

**Table:**

Refinement on *F*^2^	Primary atom site location: structure-invariant direct methods
Least-squares matrix: full	Secondary atom site location: difference Fourier map
*R*[*F*^2^ > 2σ(*F*^2^)] = 0.020	*w* = 1/[σ^2^(*F*_o_^2^) + (0.0165*P*)^2^ + 39.3966*P*] where *P* = (*F*_o_^2^ + 2*F*_c_^2^)/3
*wR*(*F*^2^) = 0.045	(Δ/σ)_max_ < 0.001
*S* = 1.16	Δρ_max_ = 2.18 e Å^−^^3^
236 reflections	Δρ_min_ = −2.22 e Å^−^^3^
15 parameters	Extinction correction: *SHELXL97* (Sheldrick, 2008)
0 restraints	Extinction coefficient: 0.00047 (4)

### Special details

**Table:**

Geometry. All e.s.d.'s (except the e.s.d. in the dihedral angle between two l.s. planes) are estimated using the full covariance matrix. The cell e.s.d.'s are taken into account individually in the estimation of e.s.d.'s in distances, angles and torsion angles; correlations between e.s.d.'s in cell parameters are only used when they are defined by crystal symmetry. An approximate (isotropic) treatment of cell e.s.d.'s is used for estimating e.s.d.'s involving l.s. planes.
Refinement. Refinement of *F*^2^ against ALL reflections. The weighted *R*-factor *wR* and goodness of fit *S* are based on *F*^2^, conventional *R*-factors *R* are based on *F*, with *F* set to zero for negative *F*^2^. The threshold expression of *F*^2^ > σ(*F*^2^) is used only for calculating *R*-factors(gt) *etc*. and is not relevant to the choice of reflections for refinement. *R*-factors based on *F*^2^ are statistically about twice as large as those based on *F*, and *R*- factors based on ALL data will be even larger.

### Fractional atomic coordinates and isotropic or equivalent isotropic displacement parameters (Å^2^)

**Table:**

	*x*	*y*	*z*	*U*_iso_*/*U*_eq_	Occ. (<1)
Ni2	0.38433 (4)	0.38433 (4)	0.38433 (4)	0.00939 (16)	
Ni3	0	0.17150 (4)	0.17150 (4)	0.01007 (16)	
Ni4	0.25	0.25	0.25	0.01036 (13)	0.106 (6)
Ni1	0	0	0	0.0092 (2)	0.349 (7)
Bi1	0.25	0.25	0.25	0.01036 (13)	0.894 (6)
Bi2	0	0	0	0.0092 (2)	0.650 (7)
B	0	0.2663 (7)	0	0.0109 (11)*	

### Atomic displacement parameters (Å^2^)

**Table:**

	*U*^11^	*U*^22^	*U*^33^	*U*^12^	*U*^13^	*U*^23^
Ni2	0.00939 (16)	0.00939 (16)	0.00939 (16)	0.00036 (13)	0.00036 (13)	0.00036 (13)
Ni3	0.0116 (3)	0.00930 (19)	0.00930 (19)	0	0	−0.00020 (16)
Ni4	0.01036 (13)	0.01036 (13)	0.01036 (13)	0	0	0
Ni1	0.0092 (2)	0.0092 (2)	0.0092 (2)	0	0	0
Bi1	0.01036 (13)	0.01036 (13)	0.01036 (13)	0	0	0
Bi2	0.0092 (2)	0.0092 (2)	0.0092 (2)	0	0	0

### Geometric parameters (Å, °)

**Table:**

Ni2—B^i^	2.133 (5)	Ni4—Ni3^xv^	2.8927 (14)
Ni2—B^ii^	2.133 (5)	Ni4—Ni3^xi^	2.8927 (14)
Ni2—B^iii^	2.133 (5)	Ni4—Ni3^x^	2.8927 (14)
Ni2—Ni2^iv^	2.4465 (15)	Ni4—Ni3^vii^	2.8927 (14)
Ni2—Ni2^v^	2.4465 (15)	Ni4—Ni3^ix^	2.8927 (14)
Ni2—Ni2^vi^	2.4465 (15)	Ni4—Ni3^viii^	2.8927 (14)
Ni2—Ni4	2.4604 (14)	Ni4—Ni3^xvi^	2.8927 (14)
Ni2—Ni3^vii^	2.6287 (13)	Ni1—Ni3^xx^	2.5648 (14)
Ni2—Ni3^viii^	2.6287 (13)	Ni1—Ni3^xxi^	2.5648 (14)
Ni2—Ni3^ix^	2.6287 (13)	Ni1—Ni3^xv^	2.5648 (14)
Ni2—Ni3^x^	2.6287 (13)	Ni1—Ni3^xxii^	2.5648 (14)
Ni3—B	2.072 (4)	Ni1—Ni3^xi^	2.5648 (14)
Ni3—B^xi^	2.072 (4)	Ni1—Ni3^xxiii^	2.5648 (14)
Ni3—Ni3^xii^	2.3480 (17)	Ni1—Ni3^xxiv^	2.5648 (14)
Ni3—Ni1	2.5648 (14)	Ni1—Ni3^xxv^	2.5648 (14)
Ni3—Ni3^xiii^	2.5648 (14)	Ni1—Ni3^xiv^	2.5648 (14)
Ni3—Ni3^xiv^	2.5648 (14)	Ni1—Ni3^xxvi^	2.5648 (14)
Ni3—Ni3^xv^	2.5648 (14)	Ni1—Ni3^xiii^	2.5648 (14)
Ni3—Ni3^xi^	2.5648 (14)	B—Ni3^xiii^	2.072 (4)
Ni3—Ni2^xvi^	2.6287 (13)	B—Ni3^xxiv^	2.072 (4)
Ni3—Ni2^xvii^	2.6287 (13)	B—Ni3^xv^	2.072 (4)
Ni3—Ni2^xviii^	2.6287 (13)	B—Ni2^xvii^	2.133 (5)
Ni4—Ni2^xvii^	2.4604 (14)	B—Ni2^xxvii^	2.133 (5)
Ni4—Ni2^xix^	2.4604 (14)	B—Ni2^xxviii^	2.133 (5)
Ni4—Ni2^xvi^	2.4604 (14)	B—Ni2^xviii^	2.133 (5)
			
B^i^—Ni2—B^ii^	110.02 (17)	Ni3^xv^—Ni4—Ni3^x^	146.645 (15)
B^i^—Ni2—B^iii^	110.02 (17)	Ni3^xi^—Ni4—Ni3^x^	116.245 (7)
B^ii^—Ni2—B^iii^	110.02 (17)	Ni3—Ni4—Ni3^x^	94.724 (4)
B^i^—Ni2—Ni2^iv^	55.01 (9)	Ni2—Ni4—Ni3^vii^	58.152 (3)
B^ii^—Ni2—Ni2^iv^	125.82 (17)	Ni2^xvii^—Ni4—Ni3^vii^	149.209 (11)
B^iii^—Ni2—Ni2^iv^	55.01 (9)	Ni2^xix^—Ni4—Ni3^vii^	58.152 (3)
B^i^—Ni2—Ni2^v^	125.82 (17)	Ni2^xvi^—Ni4—Ni3^vii^	101.320 (11)
B^ii^—Ni2—Ni2^v^	55.01 (9)	Ni3^xv^—Ni4—Ni3^vii^	116.245 (7)
B^iii^—Ni2—Ni2^v^	55.01 (9)	Ni3^xi^—Ni4—Ni3^vii^	94.724 (4)
Ni2^iv^—Ni2—Ni2^v^	90	Ni3—Ni4—Ni3^vii^	146.645 (15)
B^i^—Ni2—Ni2^vi^	55.01 (9)	Ni3^x^—Ni4—Ni3^vii^	94.724 (4)
B^ii^—Ni2—Ni2^vi^	55.01 (9)	Ni2—Ni4—Ni3^ix^	58.152 (3)
B^iii^—Ni2—Ni2^vi^	125.82 (17)	Ni2^xvii^—Ni4—Ni3^ix^	149.209 (11)
Ni2^iv^—Ni2—Ni2^vi^	90	Ni2^xix^—Ni4—Ni3^ix^	101.320 (11)
Ni2^v^—Ni2—Ni2^vi^	90	Ni2^xvi^—Ni4—Ni3^ix^	58.152 (3)
B^i^—Ni2—Ni4	108.92 (17)	Ni3^xv^—Ni4—Ni3^ix^	146.645 (15)
B^ii^—Ni2—Ni4	108.92 (17)	Ni3^xi^—Ni4—Ni3^ix^	94.724 (4)
B^iii^—Ni2—Ni4	108.92 (17)	Ni3—Ni4—Ni3^ix^	116.245 (7)
Ni2^iv^—Ni2—Ni4	125.3	Ni3^x^—Ni4—Ni3^ix^	47.89 (2)
Ni2^v^—Ni2—Ni4	125.3	Ni3^vii^—Ni4—Ni3^ix^	52.632 (19)
Ni2^vi^—Ni2—Ni4	125.3	Ni2—Ni4—Ni3^viii^	58.152 (3)
B^i^—Ni2—Ni3^vii^	153.13 (3)	Ni2^xvii^—Ni4—Ni3^viii^	58.152 (3)
B^ii^—Ni2—Ni3^vii^	95.32 (6)	Ni2^xix^—Ni4—Ni3^viii^	149.209 (11)
B^iii^—Ni2—Ni3^vii^	50.27 (13)	Ni2^xvi^—Ni4—Ni3^viii^	101.320 (11)
Ni2^iv^—Ni2—Ni3^vii^	102.978 (14)	Ni3^xv^—Ni4—Ni3^viii^	116.245 (7)
Ni2^v^—Ni2—Ni3^vii^	62.268 (15)	Ni3^xi^—Ni4—Ni3^viii^	146.645 (15)
Ni2^vi^—Ni2—Ni3^vii^	148.889 (13)	Ni3—Ni4—Ni3^viii^	94.724 (4)
Ni4—Ni2—Ni3^vii^	69.188 (17)	Ni3^x^—Ni4—Ni3^viii^	52.632 (19)
B^i^—Ni2—Ni3^viii^	50.27 (13)	Ni3^vii^—Ni4—Ni3^viii^	116.245 (7)
B^ii^—Ni2—Ni3^viii^	95.32 (6)	Ni3^ix^—Ni4—Ni3^viii^	94.724 (4)
B^iii^—Ni2—Ni3^viii^	153.12 (3)	Ni2—Ni4—Ni3^xvi^	58.152 (3)
Ni2^iv^—Ni2—Ni3^viii^	102.978 (14)	Ni2^xvii^—Ni4—Ni3^xvi^	101.320 (11)
Ni2^v^—Ni2—Ni3^viii^	148.889 (13)	Ni2^xix^—Ni4—Ni3^xvi^	58.152 (3)
Ni2^vi^—Ni2—Ni3^viii^	62.268 (15)	Ni2^xvi^—Ni4—Ni3^xvi^	149.209 (11)
Ni4—Ni2—Ni3^viii^	69.188 (17)	Ni3^xv^—Ni4—Ni3^xvi^	94.724 (4)
Ni3^vii^—Ni2—Ni3^viii^	138.28 (3)	Ni3^xi^—Ni4—Ni3^xvi^	116.245 (7)
B^i^—Ni2—Ni3^ix^	95.32 (6)	Ni3—Ni4—Ni3^xvi^	146.645 (15)
B^ii^—Ni2—Ni3^ix^	153.13 (3)	Ni3^x^—Ni4—Ni3^xvi^	116.245 (7)
B^iii^—Ni2—Ni3^ix^	50.27 (13)	Ni3^vii^—Ni4—Ni3^xvi^	47.89 (2)
Ni2^iv^—Ni2—Ni3^ix^	62.268 (15)	Ni3^ix^—Ni4—Ni3^xvi^	94.724 (4)
Ni2^v^—Ni2—Ni3^ix^	102.978 (14)	Ni3^viii^—Ni4—Ni3^xvi^	94.724 (4)
Ni2^vi^—Ni2—Ni3^ix^	148.889 (13)	Ni3—Ni1—Ni3^xx^	120
Ni4—Ni2—Ni3^ix^	69.188 (17)	Ni3—Ni1—Ni3^xxi^	180.00 (3)
Ni3^vii^—Ni2—Ni3^ix^	58.40 (2)	Ni3^xx^—Ni1—Ni3^xxi^	60
Ni3^viii^—Ni2—Ni3^ix^	108.098 (18)	Ni3—Ni1—Ni3^xv^	60
B^i^—Ni2—Ni3^x^	50.27 (13)	Ni3^xx^—Ni1—Ni3^xv^	180.00 (3)
B^ii^—Ni2—Ni3^x^	153.13 (3)	Ni3^xxi^—Ni1—Ni3^xv^	120
B^iii^—Ni2—Ni3^x^	95.32 (6)	Ni3—Ni1—Ni3^xxii^	120
Ni2^iv^—Ni2—Ni3^x^	62.268 (15)	Ni3^xx^—Ni1—Ni3^xxii^	60
Ni2^v^—Ni2—Ni3^x^	148.889 (13)	Ni3^xxi^—Ni1—Ni3^xxii^	60
Ni2^vi^—Ni2—Ni3^x^	102.978 (14)	Ni3^xv^—Ni1—Ni3^xxii^	120
Ni4—Ni2—Ni3^x^	69.188 (17)	Ni3—Ni1—Ni3^xi^	60
Ni3^vii^—Ni2—Ni3^x^	108.098 (18)	Ni3^xx^—Ni1—Ni3^xi^	120
Ni3^viii^—Ni2—Ni3^x^	58.40 (2)	Ni3^xxi^—Ni1—Ni3^xi^	120
Ni3^ix^—Ni2—Ni3^x^	53.05 (2)	Ni3^xv^—Ni1—Ni3^xi^	60
B—Ni3—B^xi^	147.8 (4)	Ni3^xxii^—Ni1—Ni3^xi^	180.00 (3)
B—Ni3—Ni3^xii^	106.08 (19)	Ni3—Ni1—Ni3^xxiii^	120
B^xi^—Ni3—Ni3^xii^	106.08 (19)	Ni3^xx^—Ni1—Ni3^xxiii^	90
B—Ni3—Ni1	73.92 (19)	Ni3^xxi^—Ni1—Ni3^xxiii^	60
B^xi^—Ni3—Ni1	73.92 (19)	Ni3^xv^—Ni1—Ni3^xxiii^	90
Ni3^xii^—Ni3—Ni1	180.00 (3)	Ni3^xxii^—Ni1—Ni3^xxiii^	120
B—Ni3—Ni3^xiii^	51.76 (8)	Ni3^xi^—Ni1—Ni3^xxiii^	60
B^xi^—Ni3—Ni3^xiii^	110.00 (12)	Ni3—Ni1—Ni3^xxiv^	90
Ni3^xii^—Ni3—Ni3^xiii^	120	Ni3^xx^—Ni1—Ni3^xxiv^	120
Ni1—Ni3—Ni3^xiii^	60	Ni3^xxi^—Ni1—Ni3^xxiv^	90
B—Ni3—Ni3^xiv^	110.00 (12)	Ni3^xv^—Ni1—Ni3^xxiv^	60
B^xi^—Ni3—Ni3^xiv^	51.76 (8)	Ni3^xxii^—Ni1—Ni3^xxiv^	60
Ni3^xii^—Ni3—Ni3^xiv^	120	Ni3^xi^—Ni1—Ni3^xxiv^	120
Ni1—Ni3—Ni3^xiv^	60	Ni3^xxiii^—Ni1—Ni3^xxiv^	120
Ni3^xiii^—Ni3—Ni3^xiv^	60	Ni3—Ni1—Ni3^xxv^	90
B—Ni3—Ni3^xv^	51.76 (8)	Ni3^xx^—Ni1—Ni3^xxv^	60
B^xi^—Ni3—Ni3^xv^	110.00 (12)	Ni3^xxi^—Ni1—Ni3^xxv^	90
Ni3^xii^—Ni3—Ni3^xv^	120	Ni3^xv^—Ni1—Ni3^xxv^	120
Ni1—Ni3—Ni3^xv^	60	Ni3^xxii^—Ni1—Ni3^xxv^	120
Ni3^xiii^—Ni3—Ni3^xv^	90	Ni3^xi^—Ni1—Ni3^xxv^	60
Ni3^xiv^—Ni3—Ni3^xv^	120	Ni3^xxiii^—Ni1—Ni3^xxv^	60
B—Ni3—Ni3^xi^	110.00 (12)	Ni3^xxiv^—Ni1—Ni3^xxv^	180.00 (3)
B^xi^—Ni3—Ni3^xi^	51.76 (8)	Ni3—Ni1—Ni3^xiv^	60
Ni3^xii^—Ni3—Ni3^xi^	120	Ni3^xx^—Ni1—Ni3^xiv^	60
Ni1—Ni3—Ni3^xi^	60	Ni3^xxi^—Ni1—Ni3^xiv^	120
Ni3^xiii^—Ni3—Ni3^xi^	120	Ni3^xv^—Ni1—Ni3^xiv^	120
Ni3^xiv^—Ni3—Ni3^xi^	90	Ni3^xxii^—Ni1—Ni3^xiv^	90
Ni3^xv^—Ni3—Ni3^xi^	60	Ni3^xi^—Ni1—Ni3^xiv^	90
B—Ni3—Ni2^xvi^	149.09 (8)	Ni3^xxiii^—Ni1—Ni3^xiv^	120
B^xi^—Ni3—Ni2^xvi^	52.37 (15)	Ni3^xxiv^—Ni1—Ni3^xiv^	120
Ni3^xii^—Ni3—Ni2^xvi^	63.474 (12)	Ni3^xxv^—Ni1—Ni3^xiv^	60
Ni1—Ni3—Ni2^xvi^	116.526 (12)	Ni3—Ni1—Ni3^xxvi^	120
Ni3^xiii^—Ni3—Ni2^xvi^	159.139 (17)	Ni3^xx^—Ni1—Ni3^xxvi^	120
Ni3^xiv^—Ni3—Ni2^xvi^	99.802 (17)	Ni3^xxi^—Ni1—Ni3^xxvi^	60
Ni3^xv^—Ni3—Ni2^xvi^	106.043 (14)	Ni3^xv^—Ni1—Ni3^xxvi^	60
Ni3^xi^—Ni3—Ni2^xvi^	60.801 (12)	Ni3^xxii^—Ni1—Ni3^xxvi^	90
B—Ni3—Ni2^xvii^	52.37 (15)	Ni3^xi^—Ni1—Ni3^xxvi^	90
B^xi^—Ni3—Ni2^xvii^	149.09 (8)	Ni3^xxiii^—Ni1—Ni3^xxvi^	60
Ni3^xii^—Ni3—Ni2^xvii^	63.474 (12)	Ni3^xxiv^—Ni1—Ni3^xxvi^	60
Ni1—Ni3—Ni2^xvii^	116.526 (12)	Ni3^xxv^—Ni1—Ni3^xxvi^	120
Ni3^xiii^—Ni3—Ni2^xvii^	99.802 (17)	Ni3^xiv^—Ni1—Ni3^xxvi^	180.00 (3)
Ni3^xiv^—Ni3—Ni2^xvii^	159.139 (17)	Ni3—Ni1—Ni3^xiii^	60
Ni3^xv^—Ni3—Ni2^xvii^	60.801 (12)	Ni3^xx^—Ni1—Ni3^xiii^	90
Ni3^xi^—Ni3—Ni2^xvii^	106.043 (14)	Ni3^xxi^—Ni1—Ni3^xiii^	120
Ni2^xvi^—Ni3—Ni2^xvii^	99.67 (3)	Ni3^xv^—Ni1—Ni3^xiii^	90
B—Ni3—Ni2^xviii^	52.37 (15)	Ni3^xxii^—Ni1—Ni3^xiii^	60
B^xi^—Ni3—Ni2^xviii^	149.09 (8)	Ni3^xi^—Ni1—Ni3^xiii^	120
Ni3^xii^—Ni3—Ni2^xviii^	63.474 (12)	Ni3^xxiii^—Ni1—Ni3^xiii^	180.00 (3)
Ni1—Ni3—Ni2^xviii^	116.526 (12)	Ni3^xxiv^—Ni1—Ni3^xiii^	60
Ni3^xiii^—Ni3—Ni2^xviii^	60.801 (12)	Ni3^xxv^—Ni1—Ni3^xiii^	120
Ni3^xiv^—Ni3—Ni2^xviii^	106.043 (14)	Ni3^xiv^—Ni1—Ni3^xiii^	60
Ni3^xv^—Ni3—Ni2^xviii^	99.802 (17)	Ni3^xxvi^—Ni1—Ni3^xiii^	120
Ni3^xi^—Ni3—Ni2^xviii^	159.139 (17)	Ni3^xiii^—B—Ni3^xxiv^	76.47 (16)
Ni2^xvi^—Ni3—Ni2^xviii^	126.95 (2)	Ni3^xiii^—B—Ni3	76.47 (16)
Ni2^xvii^—Ni3—Ni2^xviii^	55.46 (3)	Ni3^xxiv^—B—Ni3	122.2 (4)
Ni2—Ni4—Ni2^xvii^	109.5	Ni3^xiii^—B—Ni3^xv^	122.2 (4)
Ni2—Ni4—Ni2^xix^	109.5	Ni3^xxiv^—B—Ni3^xv^	76.47 (16)
Ni2^xvii^—Ni4—Ni2^xix^	109.5	Ni3—B—Ni3^xv^	76.47 (16)
Ni2—Ni4—Ni2^xvi^	109.5	Ni3^xiii^—B—Ni2^xvii^	141.71 (6)
Ni2^xvii^—Ni4—Ni2^xvi^	109.5	Ni3^xxiv^—B—Ni2^xvii^	141.71 (6)
Ni2^xix^—Ni4—Ni2^xvi^	109.5	Ni3—B—Ni2^xvii^	77.36 (3)
Ni2—Ni4—Ni3^xv^	149.209 (11)	Ni3^xv^—B—Ni2^xvii^	77.36 (3)
Ni2^xvii^—Ni4—Ni3^xv^	58.152 (3)	Ni3^xiii^—B—Ni2^xxvii^	77.36 (3)
Ni2^xix^—Ni4—Ni3^xv^	58.152 (3)	Ni3^xxiv^—B—Ni2^xxvii^	77.36 (3)
Ni2^xvi^—Ni4—Ni3^xv^	101.320 (11)	Ni3—B—Ni2^xxvii^	141.71 (6)
Ni2—Ni4—Ni3^xi^	149.209 (11)	Ni3^xv^—B—Ni2^xxvii^	141.71 (6)
Ni2^xvii^—Ni4—Ni3^xi^	101.320 (11)	Ni2^xvii^—B—Ni2^xxvii^	108.4 (3)
Ni2^xix^—Ni4—Ni3^xi^	58.152 (3)	Ni3^xiii^—B—Ni2^xxviii^	141.71 (6)
Ni2^xvi^—Ni4—Ni3^xi^	58.152 (3)	Ni3^xxiv^—B—Ni2^xxviii^	77.36 (3)
Ni3^xv^—Ni4—Ni3^xi^	52.632 (19)	Ni3—B—Ni2^xxviii^	141.71 (6)
Ni2—Ni4—Ni3	149.209 (11)	Ni3^xv^—B—Ni2^xxviii^	77.36 (3)
Ni2^xvii^—Ni4—Ni3	58.152 (3)	Ni2^xvii^—B—Ni2^xxviii^	69.97 (17)
Ni2^xix^—Ni4—Ni3	101.320 (11)	Ni2^xxvii^—B—Ni2^xxviii^	69.97 (17)
Ni2^xvi^—Ni4—Ni3	58.152 (3)	Ni3^xiii^—B—Ni2^xviii^	77.36 (3)
Ni3^xv^—Ni4—Ni3	52.632 (19)	Ni3^xxiv^—B—Ni2^xviii^	141.71 (6)
Ni3^xi^—Ni4—Ni3	52.632 (19)	Ni3—B—Ni2^xviii^	77.36 (3)
Ni2—Ni4—Ni3^x^	58.152 (3)	Ni3^xv^—B—Ni2^xviii^	141.71 (6)
Ni2^xvii^—Ni4—Ni3^x^	101.320 (11)	Ni2^xvii^—B—Ni2^xviii^	69.97 (17)
Ni2^xix^—Ni4—Ni3^x^	149.209 (11)	Ni2^xxvii^—B—Ni2^xviii^	69.97 (17)
Ni2^xvi^—Ni4—Ni3^x^	58.152 (3)	Ni2^xxviii^—B—Ni2^xviii^	108.4 (3)

Symmetry codes: (i) *y*, *z*+1/2, *x*+1/2; (ii) *z*+1/2, *x*+1/2, *y*; (iii) *x*+1/2, *y*, *z*+1/2; (iv) *x*, *y*, −*z*+1; (v) −*x*+1, *y*, *z*; (vi) *x*, −*y*+1, *z*; (vii) *x*+1/2, *y*, −*z*+1/2; (viii) *z*, *x*+1/2, −*y*+1/2; (ix) −*y*+1/2, *z*, −*x*+1/2; (x) *y*, −*z*+1/2, *x*+1/2; (xi) *z*, *x*, *y*; (xii) −*x*, −*y*+1/2, −*z*+1/2; (xiii) −*y*, *z*, −*x*; (xiv) −*z*, −*x*, *y*; (xv) *y*, *z*, *x*; (xvi) −*x*+1/2, −*y*+1/2, *z*; (xvii) −*x*+1/2, *y*, −*z*+1/2; (xviii) *x*−1/2, *y*, −*z*+1/2; (xix) *x*, −*y*+1/2, −*z*+1/2; (xx) −*y*, −*z*, −*x*; (xxi) −*x*, −*y*, −*z*; (xxii) −*z*, −*x*, −*y*; (xxiii) *y*, −*z*, *x*; (xxiv) *x*, *y*, −*z*; (xxv) −*x*, −*y*, *z*; (xxvi) *z*, *x*, −*y*; (xxvii) *x*−1/2, *y*, *z*−1/2; (xxviii) −*x*+1/2, *y*, *z*−1/2.
